# Molecular Genetics of Anthracnose Resistance in Maize

**DOI:** 10.3390/jof8050540

**Published:** 2022-05-23

**Authors:** Wendi Ma, Xinying Gao, Tongling Han, Magaji Tukur Mohammed, Jun Yang, Junqiang Ding, Wensheng Zhao, You-Liang Peng, Vijai Bhadauria

**Affiliations:** 1 Department of Plant Pathology, College of Plant Protection, China Agricultural University, Beijing 100193, China; b20203190870@cau.edu.cn (W.M.); b20203190876@cau.edu.cn (X.G.); hantongling2019@163.com (T.H.); magajimohammed2015@gmail.com (M.T.M.); yangj@cau.edu.cn (J.Y.); mppzhaws@cau.edu.cn (W.Z.); pengyl@cau.edu.cn (Y.-L.P.); 2 Ministry of Agriculture and Rural Affairs, Key Laboratory for Crop Pest Monitoring and Green Control, China Agricultural University, Beijing 100193, China; 3 College of Agronomy, Henan Agricultural University, Zhengzhou 450046, China; dingjunqiang1203@163.com

**Keywords:** anthracnose leaf blight and stalk rot, gene-for-gene diseases, hemibiotrophic pathogens, QTL, resistance genes, effectors

## Abstract

Maize (*Zea mays*), also called corn, is one of the top three staple food crops worldwide and is also utilized as feed (e.g., feed grain and silage) and a source of biofuel (e.g., bioethanol). Maize production is hampered by a myriad of factors, including although not limited to fungal diseases, which reduce grain yield and downgrade kernel quality. One such disease is anthracnose leaf blight and stalk rot (ALB and ASR) caused by the hemibiotrophic fungal pathogen *Colletotrichum graminicola*. The pathogen deploys a biphasic infection strategy to colonize susceptible maize genotypes, comprising latent (symptomless) biotrophic and destructive (symptomatic) necrotrophic phases. However, the resistant maize genotypes restrict the *C. graminicola* infection and *in planta* fungal proliferation during the biotrophic phase of the infection. Some studies on the inheritance of ASR resistance in the populations derived from biparental resistant and susceptible genotypes reveal that anthracnose is likely a gene-for-gene disease in which the resistant maize genotypes and *C. graminicola* recognize each other by their matching pairs of nucleotide-binding leucine-rich repeat resistance (NLR) proteins (whose coding genes are localized in disease QTL) and effectors (1–2 effectors/NLR) during the biotrophic phase of infection. The *Z. mays* genome encodes approximately 144 NLRs, two of which, RCg1 and RCg1b, located on chromosome 4, were cloned and functionally validated for their role in ASR resistance. Here, we discuss the genetic architecture of anthracnose resistance in the resistant maize genotypes, i.e., disease QTL and underlying resistance genes. In addition, this review also highlights the disease cycle of *C. graminicola* and molecular factors (e.g., virulence/pathogenicity factors such as effectors and secondary metabolites) that contribute to the pathogen’s virulence on maize. A detailed understanding of molecular genetics underlying the maize—*C. graminicola* interaction will help devise effective management strategies against ALB and ASR.

## 1. Introduction

Maize (*Zea mays* L.), also called corn, is one of the top three staple food crops, along with rice and wheat and is grown on more acreage than any other crop except wheat, with a current annual production of 1.16 billion metric tons (FAOSTAT 2020). Maize accounts for 20% of the calories consumed by humans daily in Africa and Mesoamerica [1]. As the global population is expected to reach approximately 9.8 billion people by 2050, it is anticipated that worldwide maize production will need to increase to an additional 1.16 billion metric tons over the current maize harvest to meet global demand based on the current consumption rate. In addition, climate change will have a negative impact on maize production in the coming decades. For example, global warming will cut cereal crop yields by 43.5 million tons for every 1 °C increase in temperature [2]. Further, climate change impacts, from rising temperatures to unpredictable rainfall patterns, will create favorable conditions for pathogens, allowing them to wreak havoc on crops, hence reducing grain yield and quality. Therefore, a 1.8% annual rate of genetic gain in grain yield will be required to meet the global demand for maize in the coming decades, in conjunction with improvements in disease resistance, agronomic performance and end-use quality. In addition to the staple food crop, maize is also used as animal feed (grain feed and silage), and a source for biofuel (e.g., bioethanol) and bio-based plastics. Approximately 40% of the maize produced in the United States is utilized in bioethanol production, and approximately 36% is fed to livestock (hogs, cattle and chickens) [3].

Fungal diseases pose a significant threat to maize production worldwide, both in terms of grain yield and quality. The crop is challenged by over a dozen adapted fungal pathogens during the growing season, causing significant economic damage, including although not limited to *Fusarium verticilliodes* (Fusarium ear and stalk rot), *F. graminearum* (Gibberella ear rot), *Cercospora zeae-maydis* (gray leaf spot), *Setosphaeria turcica* (northern corn leaf blight), *Bipolaris maydis* (southern corn leaf blight), *Cochliobolus carbonum* (northern corn leaf spot), *Colletotrichum graminicola* (anthracnose), *Diplodia maydis and D. Zeae* (Diplodia ear rot)*, Ustilago maydis* (common smut), *Puccinia polysora* (southern corn rust), *P*. *sorghi* (common rust), *Sporisorium reilianum* (head smut), *Rhizoctonia solani* (Banded leaf and sheath blight), *Alternaria alternata* (Alternaria leaf blight), *Kabatiella zeae* (eyespot), *Sphacelotheca reiliana* (head smut), *Physoderma maydis* (Physoderma leaf spot), *Phyllachora maydis* (tar spot), *Macrophomina phaseolina* (Charcoal rot) and *Aspergillus flavus* (Aspergillus ear rot) [4,5]. Recently, we reported a new fungal pathogen *Didymella glomerata*, causing Didymella leaf blight on maize in Panjin, Liaoning Province, China [6]. Yield loss attributed to these pathogens has been estimated to be 10.6% in North America [5] and 12% in Asia [7]. 

**Figure 1:**
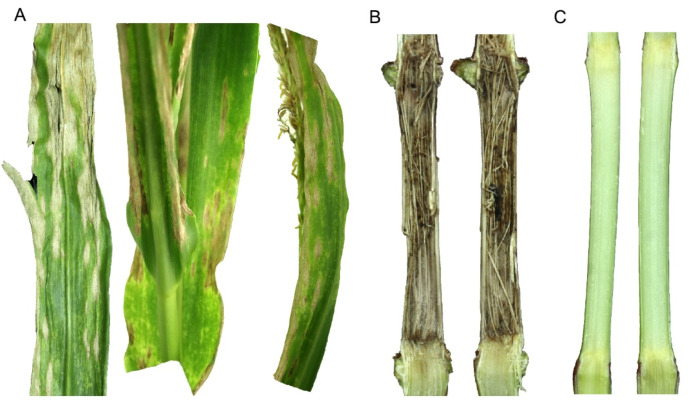
Symptoms caused by *Colletotrichum graminicola* on the susceptible *Zea mays* inbred line B73. (**A**) Gray to brown oval necrotic lesions on the B73 leaves are the typical symptom of anthracnose leaf blight. The blight lesions contain dot-like black structures called microsclerotia, which serve as the primary source of inoculum in the next growing season. (**B**) Discoloration of the pith (rotting pith) is the typical symptom of anthracnose stalk rot. The rotten pith also leads to bleaching of the upper part of the maize plants (top dieback). (**C**) Heathy pith.

**Figure 2:**
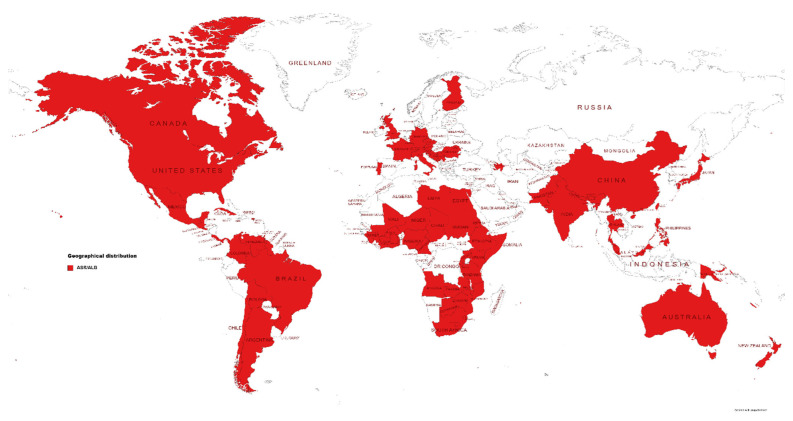
Global distribution of anthracnose leaf blight (ALB) and anthracnose stalk rot (ASR).

*C. graminicola* (Ces.) G.W. Wilson is an ascomycete fungal pathogen that mainly infects above-ground parts of maize, causing anthracnose leaf blight (ALB; Figure 1A) and anthracnose stalk rot (ASR; Figure 1B) and top dieback. The pathogen was first reported to cause severe ASR in Ohio in 1963 [8]. However, anthracnose was not considered as an economically significant disease of maize until the early 1970s, when severe epidemics wiped out the sweet corn crop in the north-central and eastern United States [9]. This sudden surge of anthracnose was perhaps attributed to the emergence of virulent races, a shift in cultural practices (e.g., widespread adoption of zero tillage or conservation tillage cropping system due to its environmental benefits) and the introduction of high-yielding cultivars lacking resistance to ALB/ASR [10]. In the 1980s and 1990s, anthracnose became a major threat to maize production and reduced grain yield by up to 40% in the corn belt of the United States, equating to a loss of more than USD 1 billion [11]. Today, ALB/ASR is considered as one of the top five most destructive diseases of maize in the United States and in Ontario, a major maize-producing province of Canada [5]. Globally, ALB/ASR is one of the top 10 most devastating diseases of maize and is prevalent in 79 countries (Figure 2). Grain yield loss due to ASR can reach up to 4.08 million metric tons annually (~1% of the total maize production) in the United States and Canada (mainly Ontario Province), which is equivalent to a direct financial loss estimated at greater than USD 562 million, not including additional losses for downgrading of the remaining crop due to poor quality. ALB can reduce grain yield by 0.03% (0.13 million metric tons) in the United States and Canada, equating to a direct financial loss of USD 17.3 million [5]. ALB/ASR, however, is an emerging disease in China, the second-largest producer of maize, and was first reported in 2018 on maize hybrids Shixing 978 and Lianchuang 808 in Huanghua City, Hebei Province, China [12]. The economic impact of the disease is yet to be determined in China.

Despite economic significance, molecular genetics underlying ASR and ALB lags behind other fungal diseases, such as ear and stalk rot caused by *Fusarium* spp. Here, we revisit the infection cycle of *C. graminicola* and discuss the genetic architecture of ALB/ASR resistance and the molecular basis of the *C. graminicola* virulence on maize.

## 2. Infection Cycle

*C. graminicola* exploits a hemibiotrophic infection strategy to colonize maize plants. The infection starts when fungal spores (conidia) land on the plant surface (e.g., leaves and stalk rind). Conidia are single-celled spores; they germinate within 6–8 h after coming into contact with the plant surface and form specialized infection structures called appressoria, which are generally sessile [13]. During conidium germination and appressorium formation, fungal germlings produce adhesive, enabling them to attach tenaciously to the plant surface. Within 15–18 h, the appressorium wall is fully melanized except at the contact point with the plant surface called the appressorial pore; the influx of water/moisture available on the plant surface into appressoria due to osmosis is believed to generate exceptionally high hydrostatic turgor, which shoots an infection peg (emerged through the appressorial) through the host plant cuticle and cell wall into the cell [10]. Once inside the cell, the infection peg differentiates into an infection vesicle, which sets the stage for *in planta* fungal infection and proliferation. The infection vesicle invaginates the host cell plasma membrane rather than puncturing it. This invaginated membrane is called the extrahyphal membrane, which is molecularly distinct from the plant cell plasma membrane. The infection vesicle gives rise to primary hyphae, which proliferate intracellularly without puncturing the host cell plasma membrane [10]. This phase of infection is called the biotrophic phase, which lasts up to 48 h in the first infected epidermal cells. However, unlike other *Colletotrichum* spp. (C. *higginsianum* [brassica anthracnose pathogen] and *C. lentis* [lentil anthracnose pathogen]), the biotrophic phase of *C. graminicola* is not confined to the first infected epidermal cells but extends into many epidermal cells and sustains at the leading edges of lesions while the centers thereof become necrotrophic, characterized by relatively thinner secondary hyphae [14]. Water-soaked lesions appear 96–120 h after infection and later turn brown with chlorotic margins. We also observed anthocyanin accumulation immediately surrounding the lesions on *Z. mays* line B73. Saucer-shaped fruiting bodies called acervuli are formed in the lesions 120 to 168 h after infection. The acervuli contain conidiogenous cells and black setae (protruding from acervuli), resembling porcupines; the conidiogenous cells give rise to conidia, which are suspended in a salmon-pink-colored mucilaginous or gelatinous matrix, which is composed of high molecular weight proline-rich glycoproteins, mycosporine-alanine and enzymes. Glycoproteins provide antidesiccant property to the matrix, enabling conidia to survive during dry conditions. Conidia in the matrix can be viable few months even at low humidity; however, conidia without being embedded in the matrix lose viability within a few hours even at high humidity. The higher concentration of mycosporine-alanine (4 µM) inhibits conidial germination in the matrix; however, when the concentration of mycosporine-alanine goes below 0.05 µM, conidia can germinate. Enzymes present in the mucilaginous matrix include cutinase and laccase. Cutinase degrades the plant cuticle, whereas laccase sequesters phenolic compounds, thereby facilitating the pathogen during penetration while protecting it from toxic phenolic metabolites present on the infection court [10]. 

Conidia are disseminated by splashing and blowing raindrops to the neighboring maize plants and thus traverse only short distances; however, dried conidial masses are dispersed to longer distances through strong wind currents. Conidia are served as a secondary source of the *C. graminicola* inoculum. ASR/ALB is, therefore, a polycyclic disease, i.e., infections can occur multiple times during the growing season. The pathogen can infect all above-ground parts of corn (e.g., crown, stalk and leaves), causing ASR and ALB (Figure 1). Infected crop debris left over after the harvest serves as a primary source of the *C. graminicola* inoculum for the following maize crop. The pathogen can survive up to 18 months on the infected crop residues; however, mycelia and conidia can only survive for a few days on soil [15]. Acervuli burst through the epidermis of the infected crop debris in the next growing season, and if maize is planted again, conidia produced in acervuli infect plants, causing ALB and ASR. ALB is prevalent between the V2 and V12 growth stages, whereas ASR and top dieback occur during physiological maturity and the R4 growth stage onwards, respectively [13]. *C. graminicola* can also infect stalk through injuries caused by stalk boring insects, such as European corn borer (*Ostrinia nubilalis*) [16]. ASR leads to top dieback in which the upper internodes and leaves are killed during grain fill, causing significant grain yield reductions [10]. Further losses can occur during harvesting due to stalk lodging caused by breakage of the lower internodes [17].

## 3. Genetic Architecture of Anthracnose Resistance in Maize

**Figure 3:**
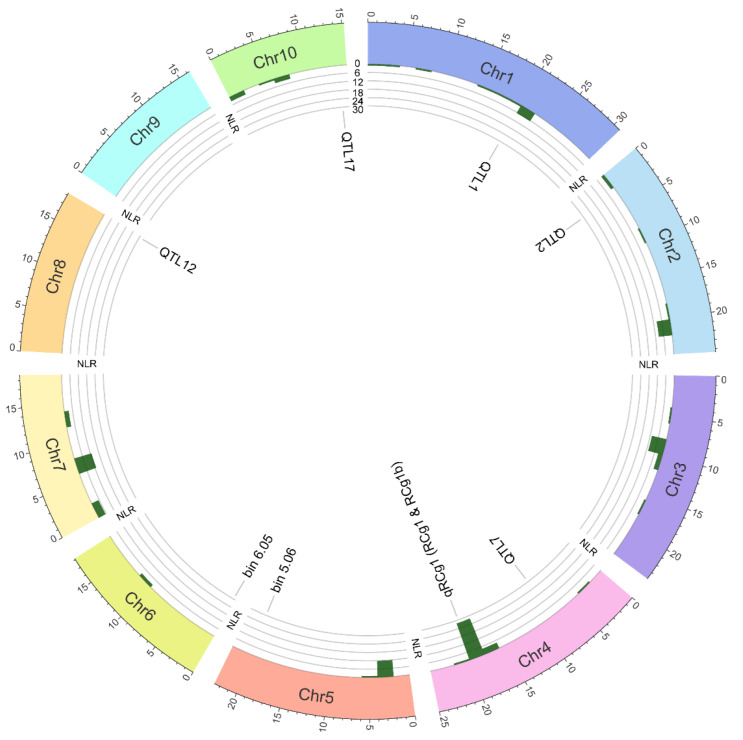
Circos plot exemplifying the distribution of 144 *NLRs* (encoding nucleotide-binding leucine-rich repeat resistance proteins) and QTL (conferring resistance to anthracnose leaf blight and stalk rot) in the *Zea mays* B73 genome. The outer track shows the *Z. mays* ideogram, comprising ten chromosomes (Chr1 through Chr10). The middle track consists of five circular ticks (6 *NLRs*/tick); the bars inside the track exhibit the frequency distribution of *NLRs* on the chromosomes. The inner track indicates the location of QTL based on their flanking marker positions listed in Table 1. The B73 genome lacks the QTL *qRCg1* controlling resistance to anthracnose stalk rot; hence, its location on the B73 genome is relative to the marker UMC15a.

In the absence of an adaptative resistance, genetic resistance in plants relies on the detection of conserved and variable pathogen molecules called, respectively, pathogen-associated molecular patterns (PAMPs) and effectors. The archetypical PAMPs include chitin (a major constituent of the fungal cell wall) and flagellin (a principal constituent of bacterial flagella). Effectors are proteinaceous and non-proteinaceous molecules that pathogens secrete into hosts to manipulate host metabolism and/or to disarm the immune system, thereby facilitating their colonization and proliferation. To detect PAMPs and effectors, plants possess a two-branched multilayered immune system. The first branch functions at the extracellular level and is called the PAMP-triggered immune (PTI) system, whereas the second branch, called the effector-triggered immune (ETI) system, acts at the intracellular level. The PTI system is activated when its components, transmembrane pattern recognition receptors (PRRs, e.g., receptor-like kinases and receptor-like proteins) detect PAMPs in the apoplast. Once the pathogens are able to disable the PTI system using their effector arsenal successfully, the ETI system comes into play, whereby nucleotide-binding leucine-rich repeat resistance proteins (NLRs, a major component of the ETI system) recognize variable effectors—that undermine both PTI and ETI systems and promote pathogen colonization—either directly through physical interactions [18], or indirectly by monitoring the integrity of guarded host virulence targets (called guardees) [19,20] or mimics thereof (called decoys) [21] in the cytoplasm. *Z. mays* (2*n* = 20) is a diploid species, whose nuclear genome (2.13 Gb) encodes ~144 NLRs (Figure 3). The number of NLRs in maize is relatively lower than those reported for other cereal crops, e.g., *Oryza sativa* (438), *Triticum aestivum* (627) and *Hordeum vulgare* (224) [22]. 

Perception of PAMPs by PRRs and effectors by NLRs triggers multilayered signaling, leading to, respectively, PTI and ETI responses in host tissues, such as oxidative (ROS) burst, Ca^2+^ influx, upregulation of mitogen-activated protein kinases, activation of pathogenesis-related genes and phytohormone synthesis [23]. The ETI system is very effective in guarding the plants against the biotrophic invaders that require living host tissue for nutrition and growth, or hemibiotrophic invaders, but not against necrotrophic invaders that kill and macerate living host tissues for nourishment and growth. In addition to the above-mentioned defense responses, ETI also induces localized cell death surrounding the infection site, called the hypersensitive cell death response (HR), which checks the ingress of the biotrophic and hemibiotrophic pathogens [24]. *C. graminicola* is a hemibiotrophic pathogen; therefore, the ETI system may underlie anthracnose resistance in maize, i.e., anthracnose is a gene-for-gene disease in which the resistant maize genotypes and *C. graminicola* recognize each other by their matching pairs of NLR protein(s) and effector(s) during the biotrophic phase of infection. If ALB/ASR is a gene-for-gene disease, it should be controlled by a single dominant resistance gene. 

Over a dozen genetic sources of anthracnose resistance have been identified in maize: LB1, LB6, ECB8, L04-2, Pa91, T111 and LB-58 (ALB-resistant lines); and A556, MP305, H21, SP288, CI88A, FR16, S11, R177 and LB31 (ASR-resistant lines). Nevertheless, genetic resistance underlying anthracnose resistance is poorly understood due to the lack of genetic studies. The first study was undertaken over 40 years ago to determine the mode of inheritance of ALB and ASR resistance in maize. Lim and White [25] generated forty-five F_1_ diallel crosses originating from ten parental inbred lines (Pa91, T111 and R177 [resistant to ALB/ASR]; Mo940, Oh07B, C123 and Va26 [susceptible to ALB/ASR]; Mo17, B73 and H95 [intermediate lines]). Analysis of ALB and ASR reactions of F1 hybrids and parental lines thereof showed that ALB and ASR were not correlated traits and that the hybrids from resistant parents were more resistant than those involving intermediate or susceptible inbred lines. In addition, ALB and ASR resistance could be combined in hybrids by crossing an ALB-resistant inbred line (Pa91 or T111) with the ASR-resistant line (R177). This implies that distinct genes in maize control ALB and ASR resistance and that the resistance is likely polygenic and additive in gene action. The quantitative nature of ASR resistance was further corroborated by the analysis of reciprocal translocation testcross populations for ASR reactions. Carson and Hooker [26] contrived 19 reciprocal translocation testcross populations and evaluated them for ASR reactions to locate chromosomal arms in the highly resistant Corn Belt inbred line A556 governing ASR resistance. These 19 reciprocal chromosomal translocation stocks were used to generate the testcross populations representing 15 out of the 20 chromosomal arms. Fourteen of the translocation stocks were in an M14 genetic background, whereas the remaining four were in the W23 genetic background. Both M14 and W23 inbred lines are highly susceptible to ASR. These nineteen stocks were crossed with A556; a susceptible tester C123 (ASR susceptible line) was also incorporated in the testcross to segregate ASR reactions and fertility/semisterility: (translocation stock ×A556) × C123. Mean differences in AR reactions between fertile and semisterile plants were used to determine which chromosomal arm confers resistance to ASR as full fertility was also a factor in enhanced resistance in addition to the chromosomal arms per se. Testcross population lines carrying the long arms of chromosomes 1, 4 and 8, and both arms of chromosome 6 showed increased ASR resistance, indicating that ASR is a polygenic trait, controlled at least by five genes. The polygenic nature of ALB was further confirmed by genetic mapping of ALB resistance in a biparental recombinant inbred line (RIL) population origination from a cross between ALB-resistant L_R_04-2 and ALB-susceptible L_S_95-1 inbred lines. Four out of seventeen QTL, one on chromosome 9 (QTL13; Table 1) and three on chromosome 10 (QTL15 through 17; Table 1 and Figure 3) were the most stable and explained 27.7 to 54.3% of the variance in ALB severity in the RIL population [27]. However, genes underlying the QTL controlling ALB resistance remain unknown.

A handful of studies suggest that anthracnose on maize is a gene-for-gene disease in which the resistance is controlled by a single dominant resistance gene or two dominant genes, one with major effect and the other with minor effect. Badu-Apraku et al. [28] evaluated F_1_, F_2_, backcross and backcross-selfed plants originating from a cross between LB-58 and A632 maize inbred lines for ALB reactions. LB-58 shows HR following the *C. graminicola* infection and thus is a highly resistant inbred line, whereas A632 is an ALB-susceptible line. All F_1_ plants were resistant to ALB, suggesting that ALB is a dominant trait. The reactions to ALB in the F_2_ population were segregated in a 3 (resistant) to 1 (susceptible) ratio both at the seedling and mature plant stages, indicating that ALB resistance in LB-58 is likely controlled by a single dominant resistance gene. The backcross progenies derived from the F_1_ × LB-58 cross were all resistant, whereas the backcross progenies resulting from the F_1_ × A632 cross were segregated for ALB reactions in a 1 (resistant): 1 (susceptible) ratio both at the seedling and mature plant stages, thereby conforming with a goodness-of-fit test for a single gene model (*p* > 0.05) for the inheritance of resistance to ALB. Likewise, the segregation of the reactions of backcross-selfed populations (BC_1_ and BC_2_) to ALB also confirmed a single dominant resistance gene (*CgL*) conferring resistance to ALB. The *CgL* gene conditions HR (characterized by chlorotic flecks) during the *C. graminicola* infection of LB-58, more likely at the biotrophic phase of infection, which restricts the pathogen from further colonization. Badu-Apraku et al. [29] also discovered a single dominant resistance gene governing ASR resistance in the ASR-resistant inbred line LB-31. The authors used a similar approach to determine the inheritance mode of ASR resistance in LB-31-derived populations. They created F_1_, F_2_ and backcross populations originating from a cross between LB-31 and ASR-susceptible inbred line B37. The F_1_ plants were all resistant to ASR; the F_2_ population was segregated for ASR reactions in a 3 (resistant) to 1 (susceptible) ratio; the progenies from F_1_ × LB-31 were all resistant, whereas the progenies from F_1_ × LB-31 were segregated for reactions to ASR in a 1 (resistant) to 1 (susceptible) ratio. The above observations imply that a single dominant gene (*CgR*) controls ASR resistance in LB-31. However, none of the two genes (*CgL* and *CgR*) have been genetically or physically mapped. In a preliminary study, Carlson [30] tracked the inheritance of ASR resistance in F_1_ and F_1_ × MP305 populations derived from ASR-resistant (MP305) and ASR-susceptible (A632) inbred lines. The majority of the lines in the populations had only one discolored internode due to ASR, similar to MP305; however, some individual plants had two to three discolored internodes, resembling A632, which showed a higher degree of susceptibility, i.e., the number of discolored internodes varied from two to five. The F_2_ population showed a continuous distribution of ASR, albeit a high degree of skewness towards fewer discolored internodes. A two-gene model could explain the variation within the F_2_ population; therefore, MP305 likely contains one major and one minor dominant resistance genes, which are likely closely linked. Toman and White [31] delved into the inheritance of ASR resistance in the populations originating from the ASR-resistant DW1035 and ASR-susceptible FRB73 inbred lines. DW1035 is derived from a cross between MP305 and FRB73, followed by repeated backcrossing (five times) with selection for ASR resistance in each cycle [26]. ASR resistance in the DW1035 × FRB73 populations (F_1_, F_2_, F_1_ × DW1035 and F_1_ × FRB73) was inherited similarly to the MP305 × A632 populations since DW1035 expressed MP305-derived ASR resistance. Generation-means analysis of the populations for the number of discolored internodes and the number of internodes >75% discolored showed that additive and dominance genetic effects contribute to ASR resistance; however, analysis in some generations revealed that a single dominant gene present in MP305 controls ASR. Taken together, the above studies showed that two closely linked genes likely control ASR resistance in maize. 

Jung et al. [32] performed inheritance analyses of ASR resistance in F_1_, F_2_, F_3_ and backcross populations originating from the DE811ASR × DE811 and DE811ASR × LH132 crosses. The inbred line DE811ASR is resistant to ASR and was developed from a cross between the recurrent parent DE811 and the resistant parent MP305, followed by backcrossing with DE811 three times (BC_3_), whereas DE811 and LH132 are susceptible to ASR. Generation-means analysis of the populations for ASR (i.e., number of discolored internodes and number of internodes >75% discolored) showed that inheritance of ASR resistance is largely additive in the populations. The authors were able to map a major QTL *qRcg1* on the long arm of chromosome 4 that conferred ASR resistance in F_2_ and F_3_ populations (Table 1 and Figure 3). *qRcg1* was flanked by two RFLP markers UMC15 and UMC66 located 12 cM apart on chromosome 4, UMC15 being closer to the QTL, explaining 21.8 and 73.2% of the variance in the ASR reactions, respectively, in the F_2_ and F_3_ populations. The authors used the Umc15 marker to select for *qRcg1* in DE811ASR and against the undesired region from MP305, thereby retaining the DE811 background as a source of European Corn Borer and avoiding genetic drag. Chung et al. [33] generated a RIL population derived from a cross between the inbred lines S11 and DK888. One to four individuals from 17 F_6_ RILs were selfed to produce 46 F_6:7_ heterogeneous inbred families (HIFs). The HIFs were phenotyped for ASR (total diseased internode area) and genotyped using SSR markers covering chromosomal regions (bins) linked with multiple disease resistance. Two bins, 5.06 and 6.05, contained QTL conferring resistance to ASR and reduced ASR by 24% and 25%, respectively; S11 contributed the resistance allele (Table 1 and Figure 3). Interestingly, these two QTL are new QTL and are distinct from *Rcg1* located on the long arm of chromosome 4 (bin 4.07) [32]. 

Broglie et al. ([34]; US Patent No. 20060223102) used the inbred line DE811ASR (BC_5_) to fine map the *qRCg1* locus. The genomes of DE811 (ASR-susceptible) and DE811ASR (ASR-resistant) are 99% identical; the introgression fragment containing *qRCg1* from MP305 represents six percent of the length of chromosome 4 [35]. Map-based cloning of *qRCg1* yielded the *RCg1* gene, which encodes an NLR protein (980 aa). Interestingly, only MP305-derived lines carry *RCg1*. The Mu lines carrying the disrupted *RCg1* were susceptible to *C. graminicola*, confirming the role of *RCg1* in ASR resistance. Furthermore, the inbred line PH09B having *qRCg1* from MP305 was resistant to ASR. To investigate the performance of *qRCg1* in the heterozygous state, as would be the case in commercial hybrid cultivars, BC3S1 (F1 [PH09B × MP305] × PH09B), plants possessing *qRCg1* and lacking *qRCg1* were used to generate testcross with the elite inbred lines PH2EJ, PH2NO, PH4CV and PH8CW. A twenty to twenty-five percent reduction in the number of internodes discolored was observed in the elite lines carrying the *RCg1* gene with that of lacking the gene; a thirty-eight to fifty-three percent reduction in the number of internodes >75% discolored in the elite lines carrying the *RCg1* gene with that of lacking the gene. Transgenic maize plants carrying the *RCg1* gene displayed ASR resistance albeit at a lower amplitude; therefore, the gene was insufficient to express ASR resistance associated with the *qRCg1* locus. Broglie and Butler [36] cloned the second gene *RCg1b* (encoding 1,428 aa NLR protein) localized in the *qRCg1* locus from DE811ASR (BC_5_) using map-based cloning. Transgenic maize plants carrying both *RCg1* and *RCg1b* expressed ASR resistance at the amplitude as conferred by the *qRCg1* locus, thereby confirming the finding of Toman and White [31] that two linked genes (one major [*RCg1*] gene and one minor [*RCg1b*] gene) are required for ASR resistance. *RCg1* and *RCg1b* are localized on the long arm of chromosome 4, which is replete with *NLR*s (34 *NLRs* on the long arm of *Z. mays* B73 chromosome 4; Figure 3).

**Table 1:** QTL in maize conferring resistance to Anthracnose stalk rot and leaf blight caused by *Colletotrichum graminicola*.

Population	Resistance Source	Population	QTL	LG	Linked Markers	Marker Interval	ASR/ALB	Reference
DE811ASR × DE811	DE811ASR (MP305)	RIL	*RCg1*	4	UMC66a-UMC15a	397.4–525.8 cM	ASR	[32]
DE811ASR × LH132	DE811ASR (MP305)	RIL	*RCg1*	4	UMC66a-UMC15a	397.4–525.8 cM	ASR	[32]
DE811ASR × DE811	DE811ASR (MP305)	NIL	*RCg1*	4	MZA2591-PHI093	61.0–63.0 cM	ASR	[34,36]
S11 × DK8883	S11	F_6:7_ HIF	*bin 5.06*	5	umc2216	518.4 cM	ASR	[33]
	S11	F_6:7_ HIF	*bin 6.05*	6	bngl2249	278.0 cM	ASR	[33]
LB58 × A632	LB58	BC	*CgL*		-	-	ALB	[28]
LB31 × B37	LB31	RIL and BC	*CgR*		-		ASR	[29]
L04-2 × L95-1	L04-2	RIL	*QTL1*	1	E32M48_308-E42M50_174	177.9–189.4 cM	ALB	[27]
	L04-2	RIL	*QTL2*	2	E35M56_680-E35M56_112	0.0–14.1 cM	ALB	
	L04-2	RIL	*QTL3*	3	E42M51_162-E42M50_76	0.0–7.6 cM	ALB	
	L04-2	RIL	*QTL4*	3	E32M48_167-E32M59_104	51.0–61.4 cM	ALB	
	L04-2	RIL	*QTL5*	4	E35M60_87-E32M60_185	0.0–10.4 cM	ALB	
	L04-2	RIL	*QTL6*	4	E32M52_73-E44M51_84	15.3–34.8 cM	ALB	
	L04-2	RIL	*QTL7*	4	Umc1511-E32M53_434	88.1–119.3 cM	ALB	
	L04-2	RIL	*QTL8*	4	E32M53_434-E44M51_135	119.3–137.7 cM	ALB	
	L04-2	RIL	*QTL9*	5	E32M48_532-E32M50_139	242.0–244.3 cM	ALB	
	L04-2	RIL	*QTL10*	8	E35M60_80-E32M50_100	0.0–23.9 cM	ALB	
	L04-2	RIL	*QTL11*	8	E32M60_94-E32M50_248	57.3–74.1 cM	ALB	
	L04-2	RIL	*QTL12*	8	E35M60_86-Phi015	85.5–107.5 cM	ALB	
	L04-2	RIL	*QTL13^S^*	9	E32M48_562-E32M48_97	126.4–157.7 cM	ALB	
	L04-2	RIL	*QTL14*	9	E32M51_314-E35M56_174	179.1–201.1 cM	ALB	
	L04-2	RIL	*QTL15^S^*	10	E32M49_698-E32M59_207	28.3–58.7 cM	ALB	
	L04-2	RIL	*QTL16^S^*	10	E32M50_118-E44M56_81	85.4–109.1 cM	ALB	
	L04-2	RIL	*QTL17^S^*	10	E32M59_76-Umc1084	161.6–191.7 cM	ALB	

## 4. Virulence/Pathogenicity Factors Contributing to the *C. graminicola* Infection on Maize

Virulence/pathogenicity factors are key for the successful infection and subsequent colonization of the plants by pathogens. These include but are not limited to effectors and secondary metabolism enzymes. 

Effectors are small secreted proteins that pathogens deliver into hosts to condition susceptibility [37]; however, these secretory proteins can be recognized by the PTI or ETI systems in resistant genotypes, hence triggering defense responses [24]. The *C. graminicola* genome likely contains 177 effector candidates, 85 of which are species-specific [38]. Vargas et al. [39] identified a set of 27 effector candidates in the *C. graminicola* genome that are likely to function in the host nucleus. One of the effectors, CgEP1, targets the host cell nucleus, wherein it binds to hundreds of sites in the maize genome. The *CgEP1* gene is differentially expressed during the infection process, with a peak at the biotrophic phase of infection. Targeted deletion of *CgEP1* led to the loss of pathogenicity of the *C. graminicola* strain lacking the gene on the susceptible maize inbred line Mo940; the knockout strain was unable to cause ALB and ASR. Chromatin immunoprecipitation followed by DNA cloning led to the identification of 58 genes, four of which were the transcription factors (TFs; viz. zinc finger, NAC, WRKY and MAD box TFs) involved in plant responses to biotic and abiotic stresses, suggesting that CgEP1 may bind to the promoters of TFs, thereby regulating various cellular processes, such as pathogenesis. The effector purportedly acts as a bacterial nucleomodulin, targeting the host nucleus in order to subvert immune responses, such as suppressing oxidative stress. A *Colletotrichum* pathogenicity-related (Cpr1) gene was identified by random mutagenesis using a restriction enzyme-mediated integration approach in *C. graminicola* [40]. This mutation was characterized as an insertion in the 3′-UTR of a gene similar to *Spc3*, which encodes one of the four essential components of the signal peptidase complex in *S. cerevisiae*, involved in the processing of signal peptides from polypeptides across the endoplasmic reticulum membrane [41,42]. The *Cpr1* mutant was comparable to the wild-type strain in various conditions *in vitro*, but it was nonpathogenic on maize stalks and leaves [40]. Cytological characterization of this mutant in intact maize leaves indicated that it was indistinguishable from the wild-type strain during spore germination, appressorium formation and penetration. However, the *Cpr1* mutant remained confined to the first infected cells and did not develop secondary hyphae; therefore, infected tissues remained symptomless [14]. It was proposed that the *Cpr1* mutant might be impaired in the secretion of one or more components required for the establishment of biotrophy and the switch to necrotrophy [40]. Eisermann et al. [43] knocked out 58 genes in *C. graminicola*, 53 of which encode effector candidates. The authors identified a cluster of five co-linear genes implicated in regulating ALB and ASR, four of which were effector candidates (CLU5a, CLU5c through CLU5e). *CLU5a* and *CLU5d* are indispensable for appressorium-mediated penetration of host cells. The Δ*CLU5a* mutants produced fully pressurized appressoria, which were defective in forming penetration pores, thereby failing to differentiate into penetration pegs. However, the Δ*CLU5a* mutants successfully penetrated the host cells through penetration pegs, which however triggered cell wall apposition around the infection sites at a higher rate than that of the wild-type strain, thereby hampering fungal virulence. However, their host targets remained unknown. 

Fungal pathogens produce a variety of secondary metabolites implicated in various functions, including pathogenicity and protection from stresses [44]. The *C. graminicola* genome contains 42 clusters of secondary metabolism genes; each cluster carries a backbone gene, such as polyketide synthases (PKS), non-ribosomal peptide synthases (NRPSs), PKS-NRPS hybrid, terpene synthases, dimethylallyl tryptophan synthases and cytochrome P450 monooxygenases [38]. The *C. graminicola PKS1* gene (*CgPKS1*) encodes type I polyketide synthase that produces tetrahydroxynaphthalene, a precursor of melanin. In fungal pathogens, such as *Magnaporthe oryzae* (the causal agent of rice blast), a melanin meshwork between the cell wall and cell membrane of appressoria enables it to generate hydrostatic turgor pressure, which is indispensable for fungal penetration. The *C. graminicola* mutant lacking *CgPKS1* displayed an albino phenotype, i.e., non-melanized mycelia and appressoria. Non-melanized appressoria of the Δ*CgPKS1* mutant failed to penetrate maize epidermal cells; therefore, the mutant was nonpathogenic on maize. In addition, the Δ*CgPKS1* appressoria were sensitive to cell wall-degrading enzymes. Melanin apposition between the cell wall and membrane of appressoria helps direct polar growth by the focal enzyme secretion, while melanin helps protect the diffusion of cell wall-degrading enzymes by improving the rigidity of the appressoria [45].

## 5. Conclusions

Anthracnose is a severe disease of maize and has the potential to wreak havoc on the maize crop. Genetic resistance coupled with cultural practices offers an environmentally friendly way to control the anthracnose disease of maize. However, widespread adoption of zero-till in cropping systems in North America will likely increase the risk of *C. graminicola* infection. Genetic resistance remains the cornerstone in controlling ALB/ASR of maize and may confer either complete or qualitative resistance mediated by a single dominant NLR gene, or incomplete or quantitative resistance controlled by multiple genes with varying levels of effects or QTLs [46,47]. It is, however, debatable whether ALB/ASR is a gene-for-gene disease in which the resistant maize genotypes and *C. graminicola* recognize each other by their matching pairs of NLR protein and effectors (1–2 effectors/NLR) during the biotrophic phase of infection. The maize genome carries approximately 144 NLR genes, only 2 of which (*RCg1* and *RCg1b*) have been cloned and functionally validated for their role in ASR resistance. Interesting, only the *RCg1* and *RCg1b* alleles from MP305- and MP305-derived lines confer ASR resistance. However, MP305 is a low-yielding, late-flowering inbred line. Therefore, only the genomic region of chromosome 4 carrying *qRCg1* should be introgressed into elite lines to avoid genetic drag, which can be achieved using marker (e.g., UMC66a, UMC14a, MZA2591 and/or PHI093)-assisted selection breeding. It would be interesting to identify the *C. graminicola* effector(s) interacting with RCg1 and RCg1b and to investigate the diversity of these effectors in the fungal populations, thereby enabling us to evaluate the durability of these resistance genes in the field. 

## Data Availability

Not applicable.
